# Incomplete bilateral duplication of the ureters identified during cytoreductive surgery for ovarian cancer: A case report

**DOI:** 10.1016/j.ijscr.2019.06.015

**Published:** 2019-06-13

**Authors:** Dimitrios Papageorgiou, Ioannis Kyriazanos, Menelaos Zoulamoglou, Eirini Deskou, Vasileios Kalles, Nikolaos Stamos, Nikolaos Ivros

**Affiliations:** Department of Surgery, Naval and Veterans Hospital of Athens, Greece

**Keywords:** Incomplete ureteral duplication, Bilateral ureteral duplication, Cytoreductive surgery, Hyperthermic Intraperitoneal Chemotherapy

## Abstract

**Introduction:**

Incomplete bilateral ureteral duplication is a very rare condition. Ureteral duplication is often asymptomatic or may be associated with several urinary tract complications.

**Case Report:**

We report a case of a 72- year- old Caucasian female who was referred to our clinic after she was diagnosed with FIGO IIIc ovarian cancer with peritoneal metastases. The patient underwent Cytoreductive Surgery plus Hyperthermic Intraperitoneal Chemotherapy. During the standard bilateral recognition of the ureters, intraoperatively, incomplete duplication of both of the ureters was identified. Bilaterally, the ureters were derived from a single renal parenchyma and duplication of the pyelocaliceal system in each kidney.

**Discussion:**

The incomplete bilateral duplication of the ureters is a rare congenital renal abnormality. In a series of 51.880 autopsies ureteral duplication was observed in 0.66% of the cases, while in another autopsy series the ureteral duplication rate was 0.68%.

**Conclusion:**

Anatomical varieties of the ureter are of utmost importance for the surgeons, because they increase the possibility of iatrogenic ureteral injury. Ureteral injuries are severe complications of pelvic operations and may increase morbidity and even cause mortality.

## Highlights

• In adults, ureteral duplication is usually asymptomatic.
• Ureteral anatomical varieties increase the possibility of iatrogenic injury.
• Ureteral injuries may increase morbidity and even cause mortality.
• If unilateral duplication is observed, bilateral duplication should be suspected.

## 1 Introduction

Malformations of the urinary tract are common congenital anomalies. One of the most common renal congenital abnormalities is ureteral duplication with an incidence that varies, among different studies, between 0.7–4%. Ureteral duplication is most common in females and may be incomplete or complete [1]. In an incomplete duplication two ureters drain into the bladder via a single common ureter. Such a duplication may be unilateral or bilateral. In a study, bilateral duplication was found in only 0.3% of the patients who underwent excretory urogram [2].

Duplication of the ureter is usually asymptomatic, but may be associated with other congenital abnormalities and with complications such as urolithiasis, urinary tract infections, vesicoureteral reflux and urinary calculi. Anatomical varieties of the ureter, also increase the possibility of iatrogenic ureteral injury, which is a potential complication of any abdominal or pelvic operation [1,3].

We report a clinical case of a patient diagnosed with ovarian cancer where an incomplete bilateral duplication of the ureters was incidentally discovered during a Cytoreductive Surgery (CRS).

The work has been reported in line with the SCARE criteria [4].

## 2 Case report

A 72- year- old Caucasian female who was diagnosed with FIGO IIIc ovarian cancer with peritoneal metastases, was referred to our department for treatment in December 2018. She was first presented in another clinic 5 months ago, with increasing pain in the pelvic region, accompanied with abdominal enlargement without weight gain. Abdomen and pelvis Computed Tomography (CT) scan showed a large left ovarian mass, abdominal ascites, peritoneal metastases and omental cake. No liver metastases were detected. The serum concentration of the CA – 125 tumor marker was elevated. Additional examinations such as thorax CT, gastroscopy and colonoscopy did not reveal any tumor or distant metastases. The cytological examination of punctured abdominal ascites revealed ovarian cancer cells.

The patient was diagnosed with FIGO stage IIIc ovarian cancer and six cycles of carboplatin and paclitaxel were planned. In December 2018 the patient underwent Cytoreductive Surgery plus Hyperthermic Intraperitoneal Chemotherapy. As part of the CRS, she underwent right hemicolectomy, rectosigmoidectomy, greater and lesser omentectomy, splenectomy, cholecystectomy, stripping of the liver capsule, as well as subdiaphragmatic peritonectomies, pelvic peritonectomies and radical hysterectomy.

**Fig. 1:**
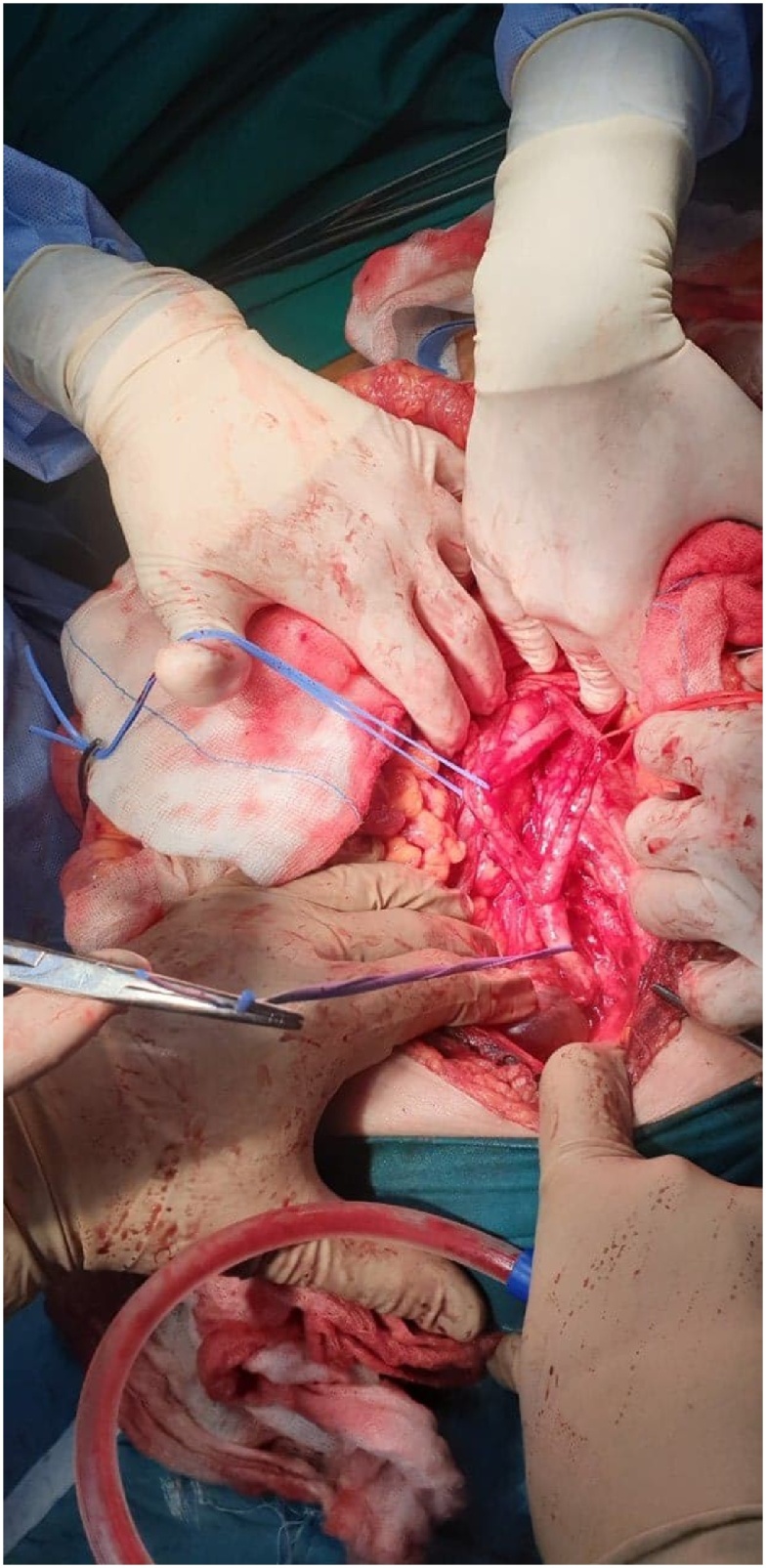
Incomplete duplication of the left ureter.

**Fig. 2:**
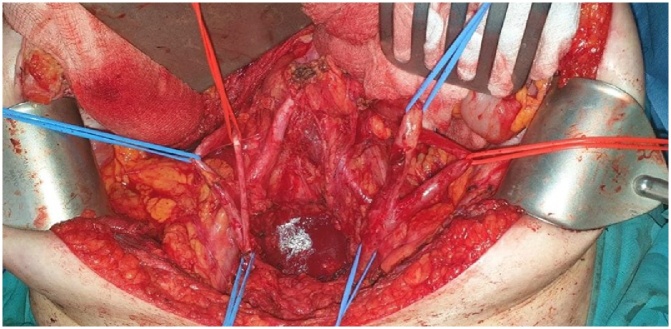
Detailed exposure of both ureters. Incomplete bilateral ureteral duplication.

The recognition and mobilization of the ureters sustain a standard procedure during CRS or any other abdominal or pelvic operation in our department. During the recognition of the left ureter, an incomplete duplication was incidentally revealed (Fig. 1). During the mobilization of the right ureter, we also detected an incomplete ureteral duplication (Fig. 2). The course of both ureters was followed centrally until the pyelocaliceal system as well as distally until their insertion into the bladder, where it was observed, that both ureters had single insertion into the bladder after they were unified 3 cm before they reach the bladder. Centrally were identified two pyelocaliceal systems in each kidney.

The operation continued uneventfully. Complete cytoreduction was achieved, and the patient received carboplatin and paclitaxel intraperitoneal, during the HIPEC procedure. Postoperatively, the patient did not present renal failure or any other urinary tract symptom and was discharged from the hospital the twenty-third day.

## 3 Discussion

The incomplete bilateral duplication of the ureters is a rare congenital renal abnormality. The reported incidence of ureteral duplication varies among different studies. The excretory urograms of 1716 children and 3480 adults revealed incomplete duplication in 57 patients (1.1%) and bilateral renal duplication in only 16 patients (0.3%). Renal duplication is twice as common in females as in males [2]. In a series of 51.880 autopsies ureteral duplication was observed in 0.66% of the cases, while in another autopsy series the ureteral duplication rate was 0.68% [5,6]. In a study of 800 cadaver kidneys procured for purposes of transplantation, only 9 cases of duplicated ureters were found, with an incidence rate of 1% [7].

Ureteral duplication is inherited as an autosomal dominant characteristic with incomplete penetrance [8]. According to the embryology, if a single ureteric bud divides before reaching the nephrogenic ridge the two buds will each stimulate the development of a separate moiety and an incomplete duplex kidney will be formed.

In adults, duplication of the ureters is usually asymptomatic, but may be associated with other complications such as urolithiasis, urinary tract infections, vesicoureteral reflux and urinary calculi. In children with ureteral duplication the risk of renal infections is 20 -times increased [9].

Anatomical varieties of the ureter, also increase the possibility of iatrogenic ureteral injury, which is a potential complication of any abdominal or pelvic operation. Iatrogenic ureteral injuries seem to be more common in gynecological and general surgical procedures, while the majority of the gynecological injuries (83%) happen during abdominal hysterectomy and salpingo – oophorectomy [3]. Thus, surgeons should be more careful, while performing hysterectomies and gynecological pelvic operations not only because of the high incidence of gynecological cancers, but also because of the higher incidence of renal abnormalities in women.

## 4 Conclussion

Ureteral injuries are severe complications of pelvic operations and may increase morbidity and even cause mortality [10]. Postoperatively recognized ureteral injuries cause more complications. For that reason, ureteral injuries should be identified and repaired intraoperatively, at occurrence, in order for the patient to have an uneventful recovery [3]. The surgeons should be aware during the recognition and mobilization of the ureters and if unilateral duplication is observed, the existence of the similar anatomic variation to the other side should be suspected.

## Declaration of Competing Interest

The authors declare no conflict of interests.

## Funding

Authors did not receive any funding for this work.

## Ethical approval

This is a case report. It is exempt from ethical approval.

## Consent

Written informed consent was obtained from the patient for publication of this case report and accompanying images. A copy of the written consent is available for review by the Editor-in-Chief of this journal on request.

## Authors' contribution

Dimitrios Papageorgiou: Operated the patient, Study conception and design, Writing the paper, Final approval.

Ioannis Kyriazanos: Operated the patient, Supervision and project administration, Review and Editing, Final approval.

Menelaos Zoulamoglou: Operated the patient, Data analysis, Review and Editing, Final approval.

Eirini Deskou: Data collection, Review and Editing, Final approval.

Vasileios Kalles: Data collection, Resources, Review and Editing, Final approval.

Nikolaos Stamos: Methodology, Data collection, Review and Editing, Final approval.

Nikolaos Ivros: Operated the patient, Review and Editing, Final approval.

## Registration of Research Studies

Not applicable.

## Guarantor

Ioannis Kyriazanos

## Provenance and peer review

Not commissioned, externally peer-reviewed.
